# *Clostridium scindens* promotes gallstone formation by inducing intrahepatic neutrophil extracellular traps through CXCL1 produced by colonic epithelial cells

**DOI:** 10.15698/mic2025.03.844

**Published:** 2025-03-19

**Authors:** Wenchao Yao, Yuanhang He, Zhihong Xie, Qiang Wang, Yang Chen, Jingjing Yu, Xuxu Liu, Dongbo Xue Xue, Wang Liyi, Chenjun Hao

**Affiliations:** 1 Department of Surgical Oncology, Jiangsu Province Hospital of Chinese Medicine, Affiliated Hospital of Nanjing University of Chinese Medicine, Nanjing 210029, China.; 2 Key Laboratory of Hepatosplenic Surgery, Ministry of Education, The First Affiliated Hospital of Harbin Medical University, Harbin, China.; 3 Department of General Surgery, The First Affiliated Hospital of Harbin Medical University, Harbin, China.; 4 Gastrointestinal Surgery Ward I, Yantai Yuhuangding Hospital, the Affiliated Hospital of Qingdao University, Yantai 264000, China.; a Equal contribution as a first author.

**Keywords:** cholelithiasis, Clostridium scindens, gut-liver axis, neutrophil extracellular traps, TLR2, CXCL1, NF-κB

## Abstract

Cholelithiasis is one of the most common diseases of the biliary system. Neutrophil extracellular traps (NETs) in the liver play an important role in accelerating the formation of gallstones. The upstream mechanism of NETs formation remains unclear. In this study, 16S rRNA sequencing was used to screen the differential gut microbiota in mice with gallstones. Transcriptome sequencing was used to screen the differentially expressed core genes and signalling pathways of *Clostridium scindens* that acted on human colonic epithelial cells. Western blotting was used to verify the protein expression of *TLR2* and the NF-κB pathway. RT-PCR was used to verify the mRNA expression of *TLR2*, *CXCL1* and the NF-κB pathway. ELISA was used to verify *CXCL1* expression in the supernatant or portal vein blood of mice. Immunofluorescence was used to detect NETs formation in cocultured neutrophils *in vitro* or in mouse livers. *Clostridium scindens* was the key differential strain in the formation of gallstones in mice. After treatment with *Clostridium scindens*, both *in vitro* and *in vivo*, the expression of *TLR2* was upregulated, the secretion of *CXCL1* was increased by regulating the NF-κB pathway. Finally, the formation of NETs and stones was significantly increased. This study reveals a new mechanism of the gut-liver immune axis in the formation of gallstones. *Clostridium scindens* acts on colonic epithelial cells through *TLR2* to regulate the NF-κB pathway and increase the secretion of *CXCL1*. *CXCL1* enters the liver via the portal vein and increases the formation of NETs in the liver, thereby accelerating gallstone formation.

## Abbreviations

LD - lithogenic diet, 

NET - neutrophil extracellular trap, 

PAMP - pathogen-associated molecular pattern. 

## INTRODUCTION

Cholelithiasis is one of the most common diseases of the digestive system, with approximately one in five adults affected, making it one of the gastrointestinal diseases with the highest socioeconomic costs 1,2. Gallstones are also a significant risk factor for acute pancreatitis, cholangitis, and gallbladder cancer 2,3, making investigating the pathogenesis of gallstones and their prevention an important research focus. The exact cause of gallstones is not fully understood, with cholesterol stones being the primary component. The main mechanisms of cholesterol stone formation include genetic susceptibility, increased hepatic cholesterol secretion, impaired gallbladder motility, rapid cholesterol crystallization, and intestinal factors 2. Among these, intestinal factors mainly refer to the influence of intestinal microbiota and their metabolites on gallstone formation through gut-liver metabolism, impacting hepatic bile acid secretion, metabolism, or composition 4,5. Notably, inflammation has also garnered increasing attention in gallstone formation. Studies have indicated that inflammation alters hepatic cholesterol and bile salt metabolism, increasing cholesterol synthesis and raising the risk of gallstone formation 6. Exploring the relationship between intestinal microbiota or their metabolites and inflammation to understand the causes of gallstone formation has become crucial.

Intestinal microbiota and their metabolites are considered triggers for various metabolic diseases such as diabetes, fatty liver disease, and obesity. Patients with gallstones often exhibit significant alterations in intestinal microbiota composition, and dysbiosis of the intestinal microbiota may be closely related to gallstone formation through its impact on bile acid and cholesterol metabolism 4. The gut-liver immune axis refers to the regulatory pathway in which gut microorganisms or their pathogenic cytokines induce an immune response in the liver, leading to disease 7. The gut-liver axis has been proven to be associated with most liver diseases 8,9. Previous research has pointed out that the gut bacterium Desulfovibrio can act as an environmental regulator, with its metabolite H2S inducing FXR expression in the liver and increasing the expression of cholesterol transporters Abcg5/g8, thereby affecting bile acid and cholesterol metabolism, which is associated with the formation of gallstones 4.

In inflammation research, neutrophil extracellular traps (NETs), as a highly specific structure of neutrophils, influence the pathological processes of various acute and chronic inflammatory diseases through their formation and degradation 10. During the formation of NETs, chromatin externalizes into a web-like structure modified by granular proteins, which act like glue, aggregating bile calcium and cholesterol crystals, which may accelerate the formation of gallstones 11. This has been identified as a novel immune factor promoting gallstone formation 12. Chen *et al*.'s research demonstrated that inhibiting neutrophil migration and NETs formation can alleviate gallbladder inflammation and reduce gallstone formation 13.

However, whether intestinal microbiota can interact with the liver to promote NETs formation and induce gallstone formation remains unclear. In this study, from the perspective of the gut-liver immune axis, we used 16S rRNA sequencing and transcriptome sequencing to identify key upstream gut bacteria involved in NETs formation, including *Clostridium scindens*, as well as immune regulatory molecules *TLR2*, *CXCL1*, and the NF-κB signaling pathway. Through *in vivo* and *in vitro* experiments, we validated the reliability of *C. scindens* stimulating colonic epithelial cells to produce *TLR2*, activating the NF-κB signaling pathway, promoting *CXCL1* expression, and inducing intrahepatic neutrophil NETosis, which may be associated with gallstone formation.

## RESULTS

### Abnormal intestinal microbiota distribution in gallstone mice

**Figure 1:**
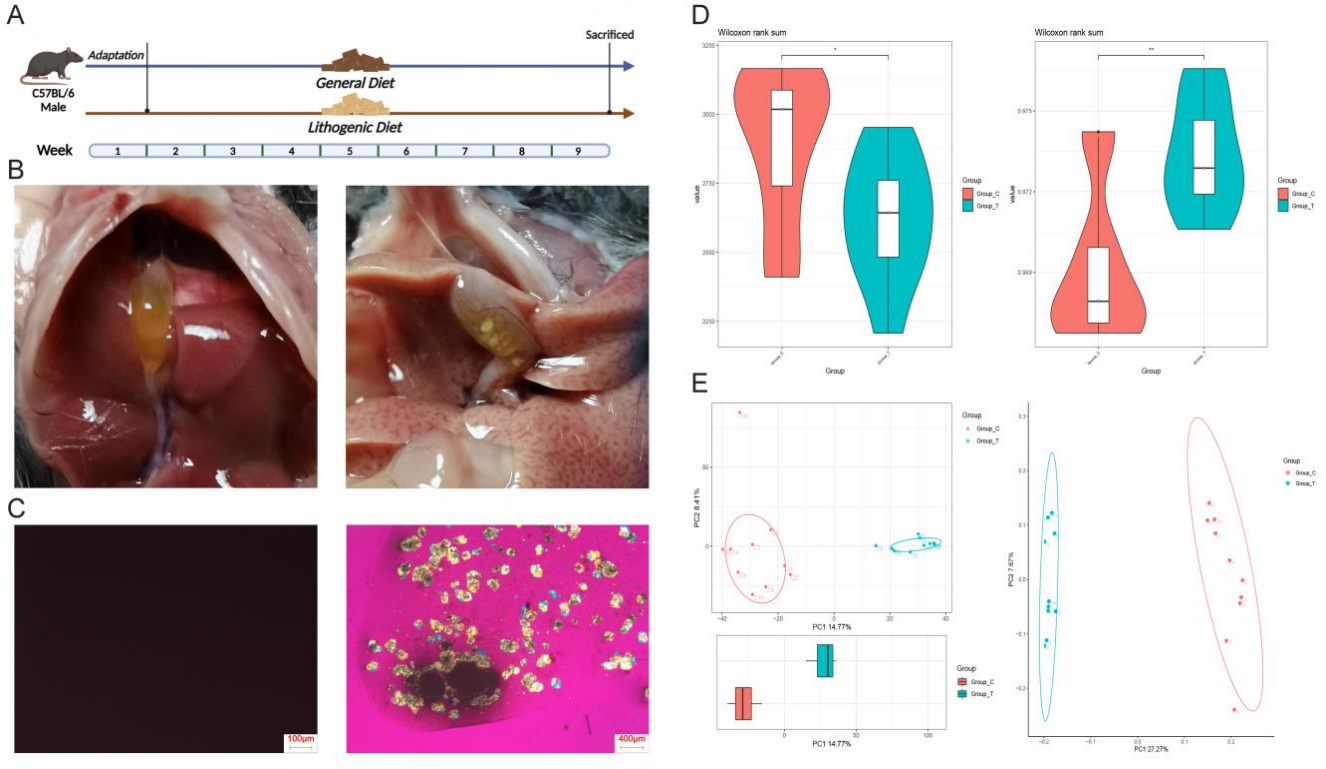
FIGURE 1: 16S rRNA sequencing was used to detect the difference of microbiota in mice with gallstones. **(A)** Mouse model construction process (Group_C, n=10; Group_T, n=10). **(B)** Anatomical pictures of mouse gallbladder; **(C)** Polarized light microscopy images of mouse bile. **(D)** Differences in α diversity of intestinal microbiota between the two groups (Group_C, n=10; Group_T, n=10). **(E)** Differences in β diversity of intestinal microbiota between the two groups (Group_C, n=10; Group_T, n=10). (Group_C: Control group, Group_T: Test group (with gallstone)).

To explore the intestinal microbiota distribution in gallstone mice, we constructed a gallstone mouse model using a lithogenic diet, with a gallstone formation rate of 8/10 in the lithogenic diet (LD) group (**Fig. 1A**). As shown in **Fig. 1B** and **C**, the gallbladder dissection images and polarized light microscopy images of bile confirm significant gallstone formation in the mice, indicating successful construction of the gallstone model. We then performed 16S rRNA sequencing on fecal samples from both groups of mice to analyze their intestinal microbiota composition and distribution. The sequencing results showed a significant reduction in intestinal microbiota abundance and α-diversity in gallstone mice (**Fig.1D**), with significant differences in β-diversity between the two groups (**Fig. 1E**). These findings indicate a notable abnormality in the intestinal microbiota distribution in gallstone mice.

### *C. scindens* is the key bacterial species in gallstone formation in mice

**Figure 2:**
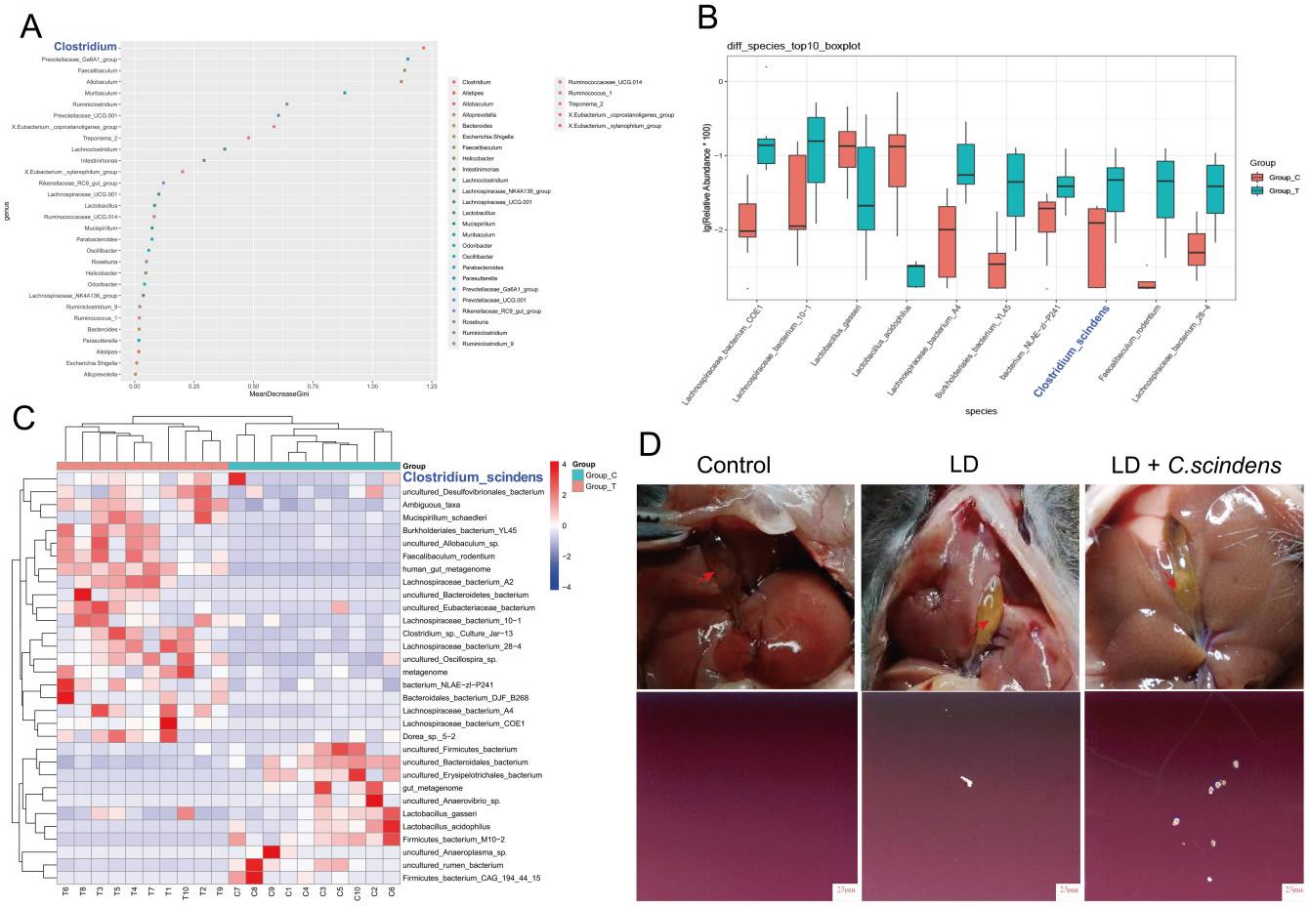
FIGURE 2: *Clostridium scindens* is the key bacterial species in gallstone formation in mice. **(A)** Dot plot of species importance from random forest analysis. The abscissa is the importance measure, and the ordinate is the species names ranked by importance. **(B)** At the species level, the top 10 species with the most significant differences between groups were identified. **(C)** At species level, there were significant differences between gallstone model mice and control mice. **(D)** Anatomical pictures of mouse gallbladder and polarized light microscopy images of mouse bile. Granular stones appeared after LD + *C. scindens* bacteria. Mice gavaged with *C. scindens* bacteria had more crystalline bile stones.

To further identify the key intestinal microbiota responsible for gallstone formation, we performed random forest analysis on the top 30 bacterial genera by relative abundance. The results indicated that the genus *Clostridium* is a key bacterial genus involved in gallstone formation (**Fig. 2A**). We then conducted Mendelian randomization analysis on 213 intestinal microbiota species to investigate their association with gallstone disease. The results showed that intestinal microbiota specifically belonging to the genus *Clostridium* was associated with gallstone disease and had the smallest p-value among all the screened genera (P=0.012) (**Table 1**). Further species-level analysis identified the top 10 most significantly different species between the two groups, with the most significantly enriched species in the *Clostridium* genus being *C. scindens* (**Fig. 2B-C**). To explore the role of *C. scindens* in gallstone formation, we performed *C. scindens* gavage experiments in mice. Both polarized light microscopy and surface-enhanced Raman spectroscopy consistently indicated that mice gavaged with *C. scindens* had higher bile crystallinity and more cholesterol crystals (**Fig. 2D**). These results confirm that *C. scindens* promote gallstone crystallization and is a key bacterial species in gallstone formation in mice.

**Table 1:** Mendelian randomization analysis of intestinal microbiota associated with cholelithiasis.

**Outcome**	**Exposure genus ID**	**pvalue**
Cholelithiasis	genus.Clostridiumsensustricto1.id.1873	0.012089887
Cholelithiasis	genus.Victivallis.id.2256	0.021153263
Cholelithiasis	genus.LachnospiraceaeUCG010.id.11330	0.039587904
Cholelithiasis	genus.Anaerostipes.id.1991	0.04619011
Cholelithiasis	genus.RuminococcaceaeUCG005.id.11363	0.047021384
Cholelithiasis	genus.FamilyXIIIAD3011group.id.11293	0.047667741

### The key mechanism by which *C. scindens* promotes gallstone formation is through inducing an increase in *TLR2* in colonic epithelial cells, activating the NF-κB signaling pathway, thereby promoting *CXCL1* expression

**Figure 3:**
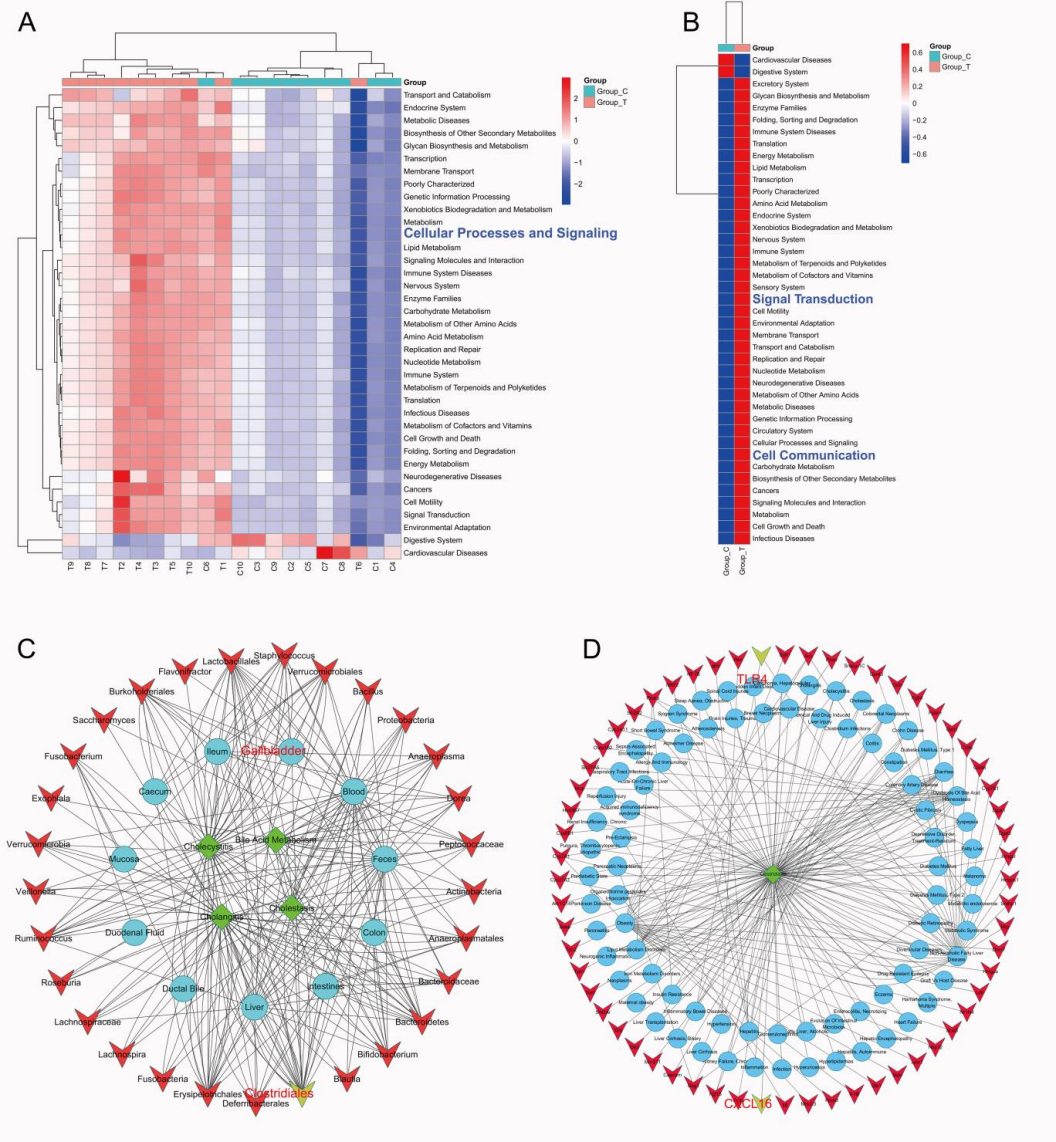
FIGURE 3: Functional enrichment analysis based on 16S rRNA sequencing, and exploration of the Amadis database. **(A-B)** Multi-group **(A)** and two-group **(B)** KEGG function prediction based on 16S rRNA sequencing. (Group_C, n=10; Group_T, n=10). **(C)** Intestinal microbiota associated with biliary tract diseases screened from Amadis database. **(D)** Amadis database to screen Clostridiales related diseases and genes.

KEGG functional analysis of the 16S rRNA sequencing data from feces of gallstone mice showed that both inter-group analysis and mean analysis (Group_T vs. Group_C) revealed a high enrichment of intercellular signal transduction and cell communication in gallstone mice (**Fig. 3A-B**). There may be a specific mode of communication between the dysbiotic intestinal microbiota and stone formation that warrants further exploration. We used the comprehensive database Amadis (http://gift2disease.net/GIFTED to screen for intestinal microbiota related to biliary diseases (**Fig. 3C**) and genes associated with *Clostridium stridiales* (**Fig. 3D**) and performed network analysis. According to the studies included in this database, *Clostridium* is associated with biliary diseases, with key downstream molecules of *Clostridium* including the CXC family and TLR family. We speculate that the mechanism of action of *C. scindens* may be related to the genes of these two families.

**Figure 4:**
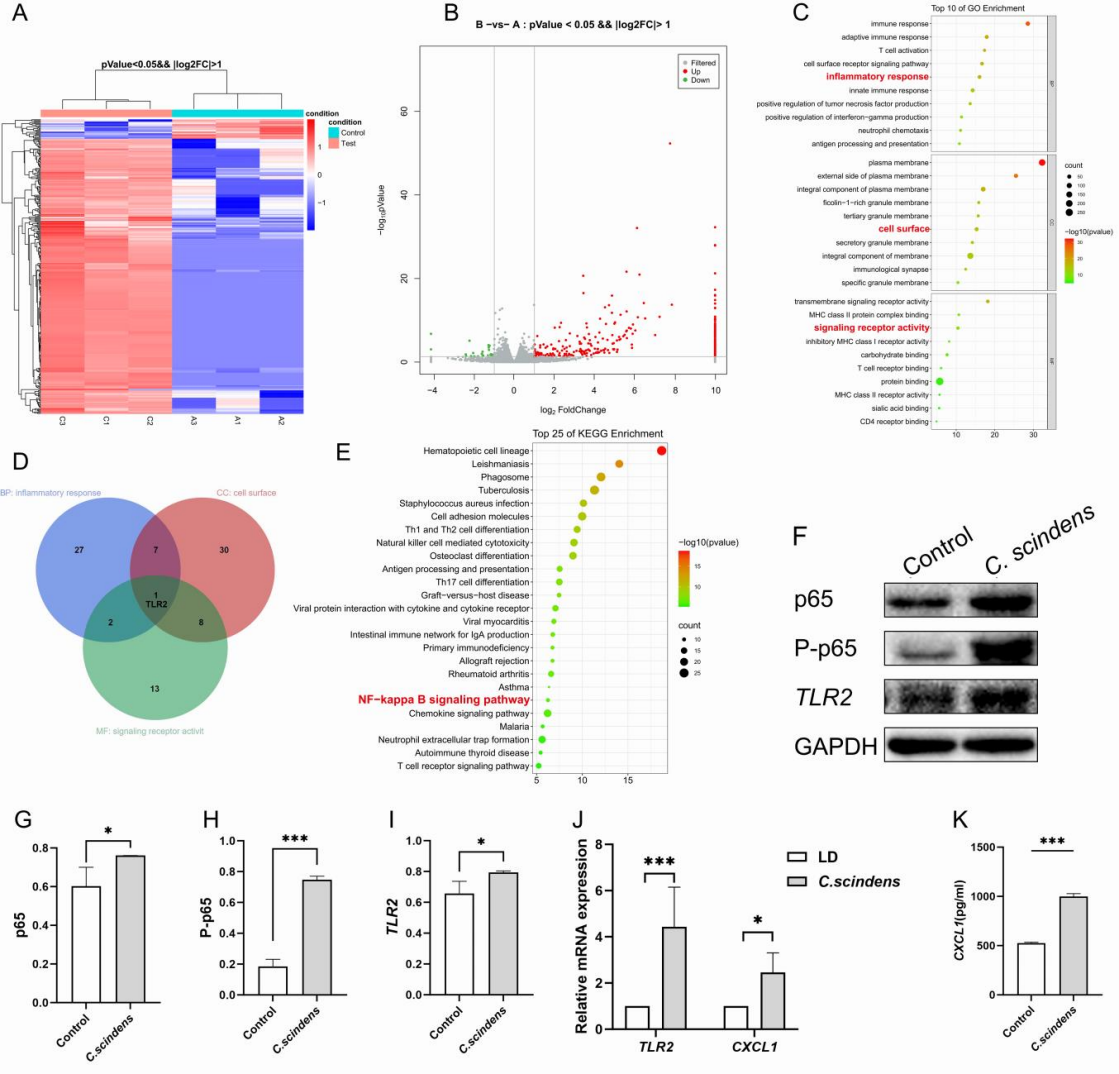
FIGURE 4: Core genes screened by mRNA-seq and verified. **(A-B) **Differentially expressed genes screened by mRNA-seq (Group A: HCoEpiC cells, n=3; Group B: C. scindens+HCoEpiC cells, n=3). **(C)** GO functional enrichment analysis, including BP, CC, MF. **(D)** Venn diagram showed that *TLR2* was the intersection gene enriched in three categories of GO. **(E)** The top 25 most significant pathways by KEGG functional enrichment analysis. **(F-I)** Western blot and bar graph analysis were used to detect the protein expression differences of p65, P-p65 and *TLR2* between the two groups. **(J)** RT-PCR was used to detect the relative expression differences of *TLR2* and *CXCL1* between the two groups. **(K)** ELISA was used to detect the *CXCL1* content in the cell supernatant, *P<0.05 **P<0.01 ***P<0.001; Group_Control, n=3; Group_C.Scindens, n=3.

Colonic epithelial cells are a crucial component of the intestinal barrier and the gut-liver axis, and they are also the cell type most closely associated with the intestinal microbiota 14,15,16. To verify our hypothesis, we co-cultured *C. scindens* with HCoEpiC cells to observe the bacterium's impact on the transcriptome of colonic epithelial cells. mRNA-seq analysis revealed that 339 genes were significantly upregulated and 24 genes were significantly downregulated in cells co-cultured with *C. scindens* (**Fig. 4A-B**, Supplementary Table 1). To explore the functions of these differentially expressed genes, we first performed GO enrichment analysis. The results showed that the genes enriched in biological processes (GO-BP), cellular components (GO-CC), and molecular functions (GO-MF) were predominantly *TLR2*, which may be a key gene (**Fig. 4C-D**). Further, KEGG enrichment analysis of the differentially expressed genes indicated significant enrichment in the NF-κB signaling pathway, which includes the cytokine *CXCL1* (**Fig. 4E**). We next examined the expression of *TLR2* and *CXCL1* in colonic epithelial cells co-cultured with *C. scindens*. The results of Western blotting showed that the expression of *TLR2* (p=0.041), p65 (p=0.049), and P-p65 (p<0.001) proteins in HCoEpiC cells significantly increased (**Fig. 4F-I**). RT-PCR results indicated that *C. scindens* significantly upregulated *TLR2* (p<0.001) and *CXCL1* (p=0.027) mRNA levels (**Fig. 4J**). In addition, ELISA results confirmed that the content of *CXCL1* in the co-culture medium significantly increased (p<0.001) (**Fig. 4K**). We further hypothesize that *TLR2*, NF-κB, and *CXCL1* may be key nodes downstream of the action of *C. scindens*.

**Figure 5:**
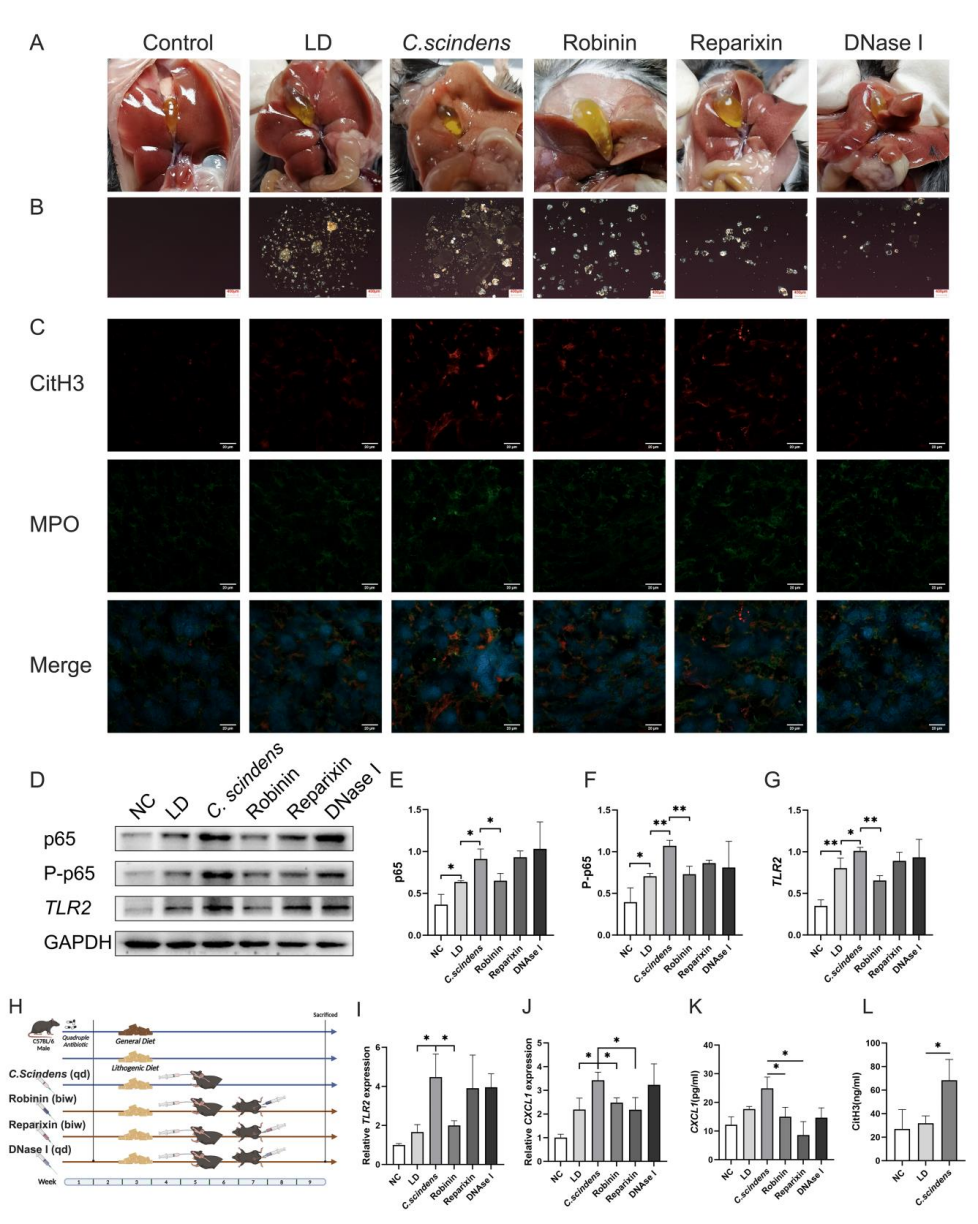
FIGURE 5: *In vivo* experiment to verify the lithogenic mechanism of *TLR2*-NF-κB-*CXCL1*-NETs-stones. **(A)** Anatomical pictures of the gallbladder of the six groups of mice; **(B)** Polarized light microscope images of bile of mice in the six groups; **(C)** The expression levels of MPO and citH3 in liver tissue of mice in the six groups were verified by immunofluorescence, thereby reflecting the production of NETs. Blue indicates DAPI, red indicates citH3 and green indicates MPO. Scale bars = 20μm; **(D-G)** Western blot and bar graph analysis were used to detect the protein expression of p65, P-p65 and *TLR2* in the six groups. **(H)** The construction process of the six groups of mouse models; **(I-J)** RT-PCR was used to verify the relative expression differences of TLR2 and *CXCL1* among the six groups. **(K)** *CXCL1* level in portal vein blood was detected by ELISA, *P<0.05 **P<0.01 ***P<0.001; NC group, n=3; LD group, n=3; C. scindens group, n=3; Robinin group, n=3; Reparixin group, n=3; DNase I group, n=3. (L) ELISA was used to detect the level of CitH3 in gallbladder bile, *P<0.05.

It is worth noting that the membrane surface receptor upstream of the NF-κB signaling pathway is *TLR2*. Therefore, we hypothesized that *C. scindens* might activate the NF-κB signaling pathway through *TLR2*, promoting *CXCL1* expression. We then established a gallstone mouse model by gavaging *C. scindens* and subsequently used Robinin to inhibit *TLR2* and Reparixin to inhibit *CXCL1* (**Fig. 5H**). Gallbladder dissection and polarized light microscopy images of bile confirmed that the *C. scindens* gavaged mice had the highest number of stones (**Fig. 5A-B**). At the same time, both inhibitors reduced stone and cholesterol crystal formation. Analysis of colonic tissue revealed that compared to the LD group, the protein expressions of *TLR2* (p=0.049), p65 (p=0.016), and P-p65 (p=0.001) were significantly increased in the *C. scindens* group. Compared to the *C. scindens* group, the Robinin group showed significantly lower protein expressions of *TLR2* (p=0.001), p65 (p=0.037), and P-p65 (p=0.007), while the Reparixin group showed no statistically significant differences (**Fig. 5D-G**). Relative mRNA expression levels indicated that compared to the LD group, the relative expressions of *TLR2* (p=0.017) and *CXCL1* (p=0.021) were significantly higher in the *C. scindens* group. After Robinin treatment, the relative expression levels of *TLR2* (p=0.023) and *CXCL1* (p=0.014) were significantly downregulated; after Reparixin treatment, only *CXCL1* (p=0.024) expression was downregulated (**Fig. 5I-J**). ELISA results confirmed that the *CXCL1* content in the portal vein blood was significantly higher in the *C. scindens* group (p=0.038), while it was significantly lower in the Robinin group (p=0.030) and the Reparixin group (p=0.010) (**Fig. 5K**).

**Figure 6:**
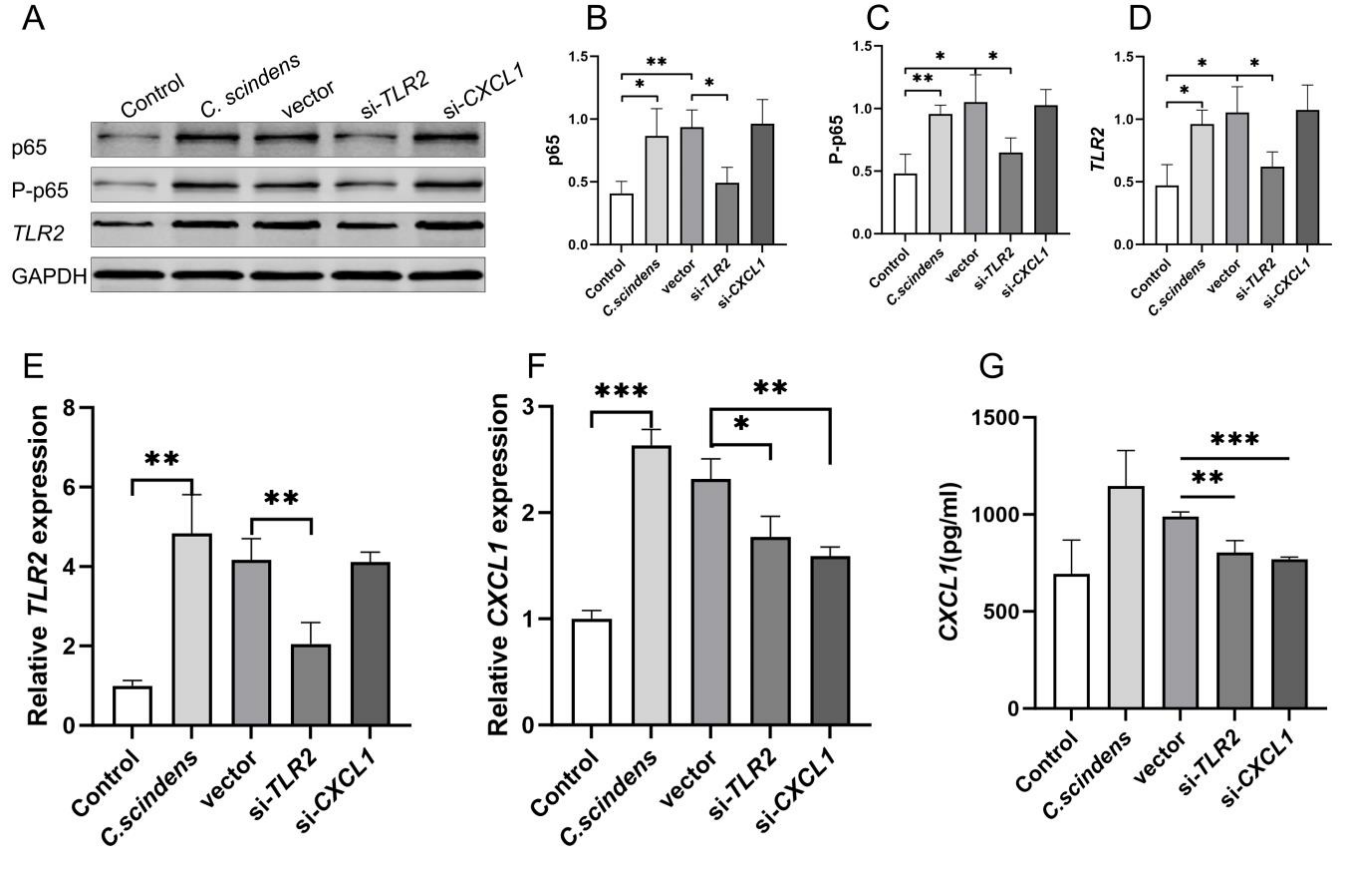
FIGURE 6: *In vitro* experiment to verify the molecular mechanism of *TLR2*-NF-κB-*CXCL1.* **(A-D)** Western blot and bar graph analysis were used to detect the protein expression of p65, P-p65 and *TLR2* in the five groups. **(E-F)** RT-PCR was used to verify the relative expression differences of *TLR2* and *CXCL1* among the five groups. **(G)** ELISA was used to verify the *CXCL1* level in the cell supernatant, *P<0.05 **P<0.01 ***P<0.001; Control group, n=3; C. scindens group, n=3; vector group, n=3; si-TLR2 group, n=3; si-CXCL1 group, n=3.

At the same time, testing of colonic epithelial cells revealed that, compared to the control group, the *C. scindens* group showed significantly increased protein expression levels of *TLR2* (p=0.013), p65 (p=0.029), and P-p65 (p=0.008) (**Fig. 6A-D**). After *TLR2* knockdown, the expression levels of *TLR2* (p=0.034), p65 (p=0.015), and P-p65 (p=0.083) proteins were significantly downregulated compared to the vector group. However, after *CXCL1* knockdown, there were no significant differences in *TLR2*, p65, and P-p65 expression (**Fig. 6A-D**). RT-PCR results showed that *C. scindens* significantly upregulated *TLR2* (p=0.003) and *CXCL1* (p<0.001) mRNA levels; after *TLR2* knockdown, *TLR2* (p=0.009) and *CXCL1* (p=0.025) were significantly downregulated compared to the vector group; after *CXCL1* knockdown, *CXCL1* was significantly downregulated (p=0.004), but there was no significant difference in *TLR2* relative expression (p=0.888) (**Fig. 6E-F**). ELISA results confirmed that the content of *CXCL1* in the *C. scindens* co-culture medium significantly increased (p=0.036), and after *TLR2* (p=0.008) or CXCL1 (p<0.001) knockdown, the content of *CXCL1* significantly decreased (**Fig. 6G**).

The above study clarified and confirmed that the key mechanism by which *C. scindens* induces stone formation is through the increased expression of *TLR2* in colonic epithelial cells, which activates the NF-κB signaling pathway and promotes the expression of *CXCL1*.

### *CXCL1* entering the liver promotes the recruitment of neutrophils and initiates gallstone formation in the form of NETosis

Muñoz LE *et al*. found that NETs are the latest discovered immune factor promoting gallstone formation, NETosis is central to gallstone formation, as it initiates the formation of gallstones, while *CXCL1* is a well-known chemokine that induces neutrophils to produce NETs 12,17. The increased *CXCL1 *from colonic epithelial cells can enter the liver through the portal vein, leading us to connect the two processes.

**Figure 7:**
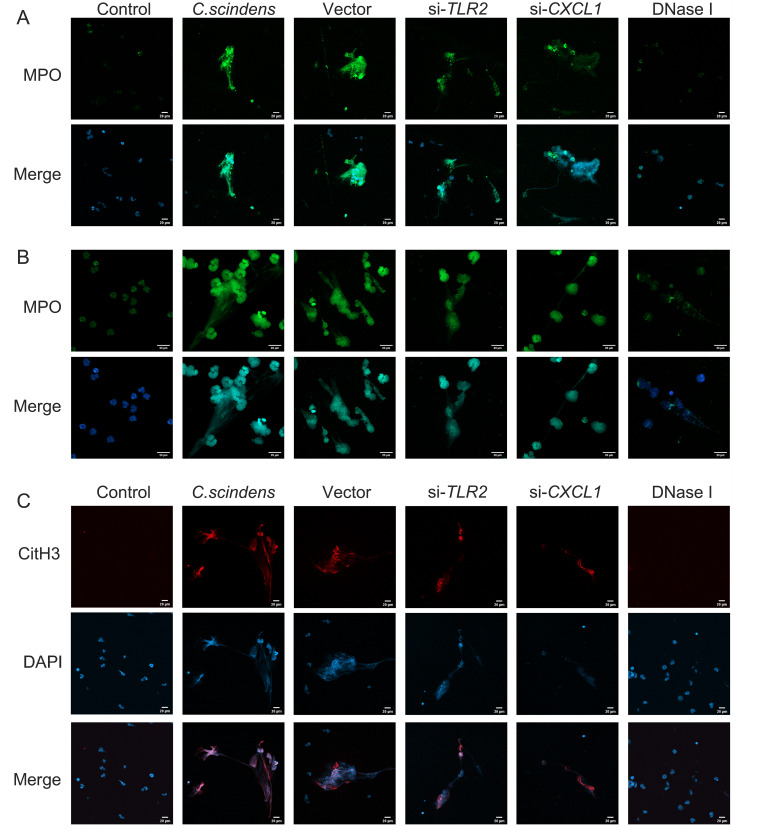
FIGURE 7: Immunofluorescence for *in vitro* validation of the TLR2-NF-κB-CXCL1-NETs molecular mechanism. **(A-B)** The expression levels of MPO in each group were verified by immunofluorescence, which reflected the production of NETs. The results of 20× magnification **(A)** and 40× magnification **(B)** were consistent. Blue indicates DAPI and green indicates MPO. Scale bars = 20μm. **(C)** The expression levels of citH3 in each group were verified by immunofluorescence, which reflected the production of NETs.

Furthermore, to verify the lithogenic mechanism of the gut-liver immune axis, we established a mouse gallstone model by gavaging *C. scindens*. The relative expression levels of NETs in the bile of mice gavaged with *C. scindens* were detected using a mouse CitH3 assay kit, and the results confirmed that the relative expression of NETs in the *C. scindens* group was significantly higher than that in the LD group (p=0.028) (**Fig. 5L**). Building on the use of previous inhibitors, DNase I was applied to inhibit NETs. Anatomical images of the mouse gallbladder and polarized microscopy images of bile confirmed that both inhibitors reduced the formation of stones and cholesterol crystals (**Fig. 5A-B**). The formation of NETs was compared by analyzing the staining intensity of citH3 and MPO in mouse liver tissues, with the *C. scindens* group showing the highest NETs formation, which was significantly reduced after using inhibitors (**Fig. 5C**). This trend was consistent with gallstone formation. Additionally, gallbladder anatomical images, polarized microscopy of bile, PCR, WB, and ELISA results all indicated that DNase I inhibition only reduced NETs formation and stone formation, without affecting the expression of *TLR2*, p65, P-p65, and *CXCL1*. Subsequently, based on co-culturing *C. scindens* and HCoEpiC cells, neutrophils were cultured in the upper chamber of a transwell to simulate the stone-forming environment of the gut-liver axis, and DNase I was used to inhibit NETs formation. Immunofluorescence results of neutrophils showed that, at both 20x (**Fig. 7A**) and 40x magnifications (**Fig. 7B**), NETs formation was most evident in the co-culture and vector groups. After down regulating *TLR2* and *CXCL1*, NETs formation was significantly reduced; the control and DNase I groups had the lowest levels of NETs formation. As a key marker of NETs generation, histone H3 (citH3) staining results were consistent with MPO staining results (**Fig. 7C**).

The above results confirm that *CXCL1* promotes the increased recruitment of neutrophils in mice with gallstones and initiates gallstone formation through NETosis. Combined with previous findings, this establishes a novel lithogenic mechanism of the gut-liver axis: *C. scindens* activates the NF-κB signaling pathway via *TLR2*, promoting the secretion of *CXCL1*, which enters the liver and stimulating neutrophils to form NETs, ultimately leading to gallstone formation.

## DISCUSSION

As a disease closely related to inflammatory responses, the etiology of gallbladder stones is a scientific issue deserving thorough investigation. This study innovatively connects the intestinal microbiota with gallbladder stones, focusing on the gut-liver immune axis. By using various sequencing methods combined with *in vivo* and *in vitro* experiments, it proposes and verifies that *C. scindens* induces the production of *CXCL1* by colonic epithelial cells, which in turn stimulates an increase in NETs in liver neutrophils, promoting the formation of gallbladder stones.

Changes in the intestinal microbiota and imbalances in gut microbiome homeostasis are closely related to the formation of gallbladder stones 18. In this study, through 16S rRNA sequencing, we found that the abundance of *C. scindens* in the intestinal microbiota of mice with gallstones increased. It has emerged as a key bacterium involved in the formation of gallbladder stones in mice. Sequencing and KEGG enrichment analysis also confirmed that this bacterium is involved in signal transduction and cell communication. *C. scindens* is an anaerobic bacterium belonging to the *Bacillaceae* family. Although it is usually present in low abundance in the gut, its presence is crucial for maintaining gut microbial balance and bile acid metabolism. It has 7α-dehydroxylase activity, and when its abundance increases, secondary bile acid synthesis also rises. This increase suppresses other intestinal microbiota and triggers feedback regulation in the liver, leading to increased cholesterol secretion 19,20. Previous studies have shown that increased *C. scindens* in the gut alters bile acid metabolism through its bai operon-encoded genes and promotes primary sclerosing cholangitis associated with inflammatory bowel disease 21. Moreover, *C. scindens* has been found to activate downstream inflammatory and immune responses. This bacterium can promote the antiviral effects of type I interferons through the TLR7/MyD88 signaling pathway in dendritic cells 22. In a mouse model colonized by *C. scindens*, the expression of monocytes in the bone marrow is significantly elevated, and a strong neutrophil response is generated when the gut receives external stimuli 23.

As the first checkpoint for regulating the gut-liver axis, the role of colonic epithelial cells is crucial. In our study, transcriptomic differential gene enrichment analysis of *C. scindens* co-cultured with HCoEpiC cells pointed to *TLR2*. Further KEGG functional analysis of differential genes highlighted the NF-κB signaling pathway, a classic inflammatory pathway. We validated the expression levels of *TLR2*, the NF-κB signaling pathway, and *CXCL1* in colonic epithelial cells after co-culture through *in vitro* experiments. Western blot, PCR, and ELISA experiments showed that the mRNA and protein levels of *TLR2*, p65, P-p65, and *CXCL1* were significantly upregulated. Thus, we established the theoretical foundation that *C. scindens* stimulates colonic epithelial cells' *TLR2* to activate the NF-κB pathway, leading to increased levels of the inflammatory chemokine *CXCL1* in the gut-liver axis.

Colonic epithelial cells possess immune functions and can participate in local immune responses by secreting cytokines and chemokines 24. A dysregulated gut microenvironment can alter the function of colonic epithelial cells. Previous studies indicated that *Fusobacterium nucleatum* and *Haemophilus parainfluenzae* can induce tumor formation in the gut by modulating DNA methylation in colonic epithelial cells 25. An increase in anaerobic bacteria interacts with TLR2 and TLR4 on colonic cells, raising reactive oxygen species levels and promoting cholesterol synthesis and cell proliferation 26. As a membrane receptor, TLR2 recognizes and binds to a variety of pathogen-associated molecular patterns (PAMPs), activating downstream signaling pathways and triggering the production of inflammatory mediators to initiate the immune response 27. TLR2, a key member of the Toll-like receptor family, recognizes PAMPs such as peptidoglycans or lipoproteins from intestinal microbiota, activating the NF-κB signaling pathway and inducing downstream pro-inflammatory cytokines and chemokines 28. Robinin has been shown to ameliorate oxidized low-density lipoprotein-induced inflammatory insult through the TLR/NF-κB pathway 29, which is why it was chosen as the intervention in this study.

The activation of *TLR2* by the NF-κB pathway, leading to the production of downstream pro-inflammatory cyto-kines and chemokines, has been well established 30. Liu *et al*. pointed out that *Lactobacillus reuteri* and *Escherichia coli* activate the NF-κB signaling pathway via *TLR2*, promoting arylalkylamine N-acetyltransferase, revealing a specific mechanism by which the intestinal microbiota regulates host melatonin production 31. *CXCL1* is a chemokine downstream of the NF-κB signaling pathway 32,33. Research has shown that the NF-κB pathway can upregulate the pro-inflammatory chemokine *CXCL1*, leading to mitochondrial dysfunction, inflammation, and cell migration, thereby promoting disease progression 34, consistent with our findings. Besides, reparixin is a noncompetitive allosteric inhibitor of the chemokine receptors CXCR1 and CXCR2 activation with IC50 values of 1 and 100 nM, respectively. It has been shown to inhibit *CXCL1* expression in a variety of mouse models, thereby reducing inflammatory responses 35,36.

Activated neutrophils can release a net-like DNA structure encased with various intracellular substances, including elastase, cathepsin G, and myeloperoxidase, known as neutrophil extracellular traps (NETs). As a highly specific structure of neutrophils, the generation and degradation of NETs can influence the pathological processes of various acute and chronic inflammatory diseases 10. NETs are a recent immune factor promoting gallbladder stone formation. The chromatin externalizes into a network structure modified by granular proteins, which act like "glue" to aggregate bile calcium and cholesterol crystals, thereby accelerating gallstone formation 11. Luis E Muñoz *et al*. reaffirmed in Immunity that NETs are a new immune factor promoting gallbladder stone formation, and inhibiting NETs formation or depleting neutrophils can effectively reduce gallstone growth 12.

Furthermore, *CXCL1* is well-recognized for its role in recruiting large numbers of neutrophils, leading to NETs formation that exacerbates inflammatory processes and promotes disease progression 37. We hypothesize that increased *CXCL1* secretion by colonic epithelial cells, upon entering the liver through the portal vein, recruits and stimulates neutrophils to produce NETs, thereby promoting stone formation. To further investigate this, we co-cultured *C. scindens* with colonic epithelial cells and used a transwell chamber to culture neutrophils in the upper chamber, simulating the environment of distal organs.

Immunofluorescence experiments first confirmed that the highest amount of NETs was produced in the co-culture group, with a reduction in NETs production observed after down regulating *TLR2* or *CXCL1*. The expression levels of *TLR2*, *CXCL1*, p65, and P-p65 were significantly upregulated at both protein and mRNA levels in the co-culture group. Additionally, down regulating *TLR2 *and *CXCL1* confirmed that *TLR2*, as an upstream core gene, regulates the NF-κB signaling pathway and the expression of *CXCL1*. Subsequent *in vivo* experiments also confirmed increased NETs and gallstone formation in mice gavaged with *C. scindens*, consistent with the *in vitro* findings.

Previous research has shown that in the liver, *CXCL1* derived from hepatic sinusoidal endothelial cells can recruit and generate NETs, promoting microthrombus formation in the liver sinusoids and exacerbating portal hypertension 38. *CXCL1*, as a chemokine, not only induces inflammation in surrounding tissues but can also reach distal organs to recruit neutrophils and trigger inflammatory responses 34. Antipenko S *et al*. reported that increased expression of the chemokine *CXCL1* promotes neutrophil chemotaxis and increases NETs in circulation, thereby exacerbating heart failure 39. Additionally, a recent study found that upregulation of *CXCL1* gene expression significantly recruits tumor-associated neutrophils, leading to NETs formation and promoting liver cancer metastasis 40.

This study also has certain limitations. We have only verified the role of a single bacterium in gallstone formation. However, other microbiota may also play a similar role in promoting NETs formation, thereby facilitating stone formation. Therefore, our study only provides one aspect of the mechanism, and further comprehensive exploration of the formation mechanisms of gallstones is still urgently needed.

In conclusion, this study demonstrates that *C. scindens* activates the NF-κB signaling pathway by acting on the pattern recognition receptor (TLR2) in colonic epithelial cells, leading to an upregulation of downstream *CXCL1* expression. *CXCL1* enters the liver tissue via the portal vein, recruiting neutrophils and generating excessive NETs. These NETs serve as scaffolds for cholesterol crystals, accelerating stone formation.

## MATERIAL AND METHODS

### Cell and bacterial culture

*C. scindens* strain ATCC 35704, which was purchased from American Type Culture Collection (ATCC, Manassas, VA, USA), was cultured in enhanced *Clostridium* culture medium at 37°C under anaerobic conditions.

Cultured human normal colon epithelial HCoEpiC cells (C1228, WHELAB, Shanghai, China), and *C. scindens* and cells were cocultured under anaerobic conditions (5% CO_2_, 5% O_2_), the ratio of cells to bacteria was 1:10, the coculture used complete medium, and the cells of each group were collected after 2 h of anaerobic coculture. The *C. scindens* cells in the 2 h anaerobic culture were aspirated and then cocultured with neutrophils in a transwell chamber under conventional conditions. The medium used for coculture was complete medium. Neutrophils were cultured in the upper chamber, and *C. scindens* and HCoEpiC cells were cultured in the lower chamber (the ratio of cells to colonies was 1:10). The upper cell volume was 5*10^5^/well, and the lower cell volume was 4.5*10^5^/well. After culturing for 24 h, HCoEpiC cells and neutrophils were collected for subsequent experiments.

### Mice

All experimental protocols were approved by the Ethics Committee of Harbin Medical University. Adult male C57BL/6J mice (3 weeks old, Liaoning Changsheng Biotechnology Co., Ltd, China) were housed in a controlled environment (12 h light-dark cycle) in terms of temperature (18-24°C) and humidity (50-60%). Each group was fed the indicated diet and water ad libitum. The mice were given 1 week to adapt to the new environment, followed by 8 weeks of dietary intervention.

Sixty mice were randomly divided into a negative control group (NC group), lithogenic diet group (LD group), lithogenic diet + bacteria irrigation group (*C. scindens* group), lithogenic diet + bacteria irrigation + robinin group (Robinin group), lithogenic diet + bacteria irrigation + reparixin group (Reparixin group), and lithogenic diet + bacteria irrigation + DNase I group (DNase I group). The NC group was fed a normal diet, and the other five groups were fed a lithogenic diet (LD, containing 1.25% cholesterol and 0.5% cholic acid). Robinin (301-19-9, MCE, China) is present in the flavonoid fraction of Vigna unguiculata leaves. Robinin was used to inhibit the upregulated expression of *TLR2*. Robinin ameliorates oxidized low-density lipoprotein-induced inflammatory insult through the TLR/NF-κB pathway 29. Reparixin was purchased from Yuanye Biotechnology Company (266359-83-5, Yuanye, Shanghai, China) to inhibit the upregulated expression of *CXCL1*. Reparixin is a noncompetitive allosteric inhibitor of the chemokine receptors CXCR1 and CXCR2 activation with IC_50_ values of 1 and 100 nM, respectively. It has been shown to inhibit *CXCL1* expression in a variety of mouse models, thereby reducing inflammatory responses 35,36. DNase I (11284932001, Roche, Germany) is a double-stranded specific endonuclease that degrades DNA. The protein is composed of two central β-folds, each consisting of six β-strands. It has been widely used in *in vivo* and *in vitro* studies of NETs 41,42. Intraperitoneal injection of DNase I effectively inhibited the production of NETs in mice 43,44.

Microflora intervention: Gavage was carefully administered to animals immobilized using a gavage needle suitable for mice. *C. scindens* at 1 × 10+ CFU was administered by gavage every day until sacrifice, and the mice in the negative control group were gavaged with PBS only.

Drug intervention: The NC group was not administered; the LD group received intraperitoneal injection of the same amount of PBS; the Robinin group received intraperitoneal injection of Robinin twice a week, each time each mouse was administered 6 mg/kg; the Reparixin group was given an intraperitoneal injection of Reparixin twice a week, each time each mouse was given 25 mg/kg; and the DNase I group was given a daily intraperitoneal injection of DNase I, each time each mouse was given 5 mg/kg.

After 8 weeks of dietary, microbiota, and drug interventions, stools were collected every day at 7 am. We stimulated the end of the mouse rectum with a cotton swab to facilitate defecation, collected mouse faeces with a sterile tube, and immediately refrigerated it in a -80°C freezer. Mice were euthanized after an 8-hour fast. Liver and colon tissue samples and portal blood were collected from mice and frozen at -80°C for 10 minutes. Meanwhile, to observe the effect of LD-induced gallstone phenotype, gallstone formation was evaluated macroscopically, and gallbladder bile was examined using a cover-free polarized light microscope.

### Intestinal microbiota 16S rRNA sequencing and data analysis

The total genomic DNA of samples was extracted using the MagPure Soil DNA LQ Kit (Magen, Guangzhou, China). The concentration of DNA was verified with a NanoDrop (Thermo Fisher, United States) and agarose gel electrophoresis. Bacterial DNA was amplified with primers targeting the V3-V4 regions (50-TACGGRAGGCAGCAG-30, 50-AGGGTATCTAATCCT-30). Amplicon quality was visualized using gel electrophoresis, purified with AMPure XP beads (Agencourt), and amplified for another round of PCR (Bio-Rad, United States). After purification with AMPure XP beads again, the final amplicon was quantified using a Qubit dsDNA assay kit. Equal amounts of purified amplicons were pooled for subsequent sequencing by the Illumina MiSeq platform by OE Biotech.

Data analysis Operational taxonomic unit (OTU) analysis clustered sequences at the 97% similarity level (USEARCH, version 10.0), and 0.005% of all sequences was used as the threshold to filter OTUs. Species annotation and taxonomic analysis: The 16S rRNA:Silva rRNA gene database was selected (Release128, http://www.arb-silva.de 45. Alpha index analysis: Shannon diversity curves and rank species abundance (RSA) curves (rank abundance curves) were used 46. The software for these tasks was Mothur version v.1.30 (http://www.mothur.org . Beta diversity: The species diversity matrix was calculated by a variety of algorithms, such as binary Jaccard, Bray Curtis, and unweighted UniFrac (only for bacteria). The principal coordinate analysis (PCoA) results were plotted using R software. Line discriminant analysis (LDA) effect size (LEfSe) analysis (http://huttenhower.sph.harvard.edu/lefse , the analysis of significant differences between groups (which can be called biomarker analysis), used LDA to estimate the impact of the abundance of each component (species) on the differences, and a logarithm of LDA score of 4.0 was set as the cut-off for significant differences. KEGG pathways and COG (clusters of orthologous groups) functions were analysed using PICRUSt software 47,48. To determine if taxonomic differences in the microbiota could be used to classify samples into different cohorts and a machine learning algorithm named random forest (RF) was applied to analyse the genus-level abundances of gut bacteria by the R package randomForest 49.

### Amadis database

Amadis (http://gift2disease.net/GIFTED is a database that provides experimentally supported microbiota-disease associations 50. With the aid of Amadis's network analysis tools, we found that there could be an association between the CXCL/TLR family, Clostridiales and biliary tract disease.

### Mendelian randomization analysis

The gut microbiome data were obtained from the MiBioGen database (https://mibiogen.gcc.rug.nl/), with a total of 213 gut microbiome datasets downloaded. The gallstone data came from the Finnish database R11 (https://r11.finngen.fi/pheno/K11_CHOLELITH). The data downloaded from both databases were processed using RStudio. We used the gut microbiome data as the exposure and the gallstone disease data as the outcome. Subsequently, Mendelian randomization analysis was performed using the TwoSampleMR package, with a p-value < 0.05 considered statistically significant, indicating a correlation between them.

### Western blot

The fold changes in *TLR2*, phospho-p65 (P-p65) and p65 protein expression were detected by Western blot. Protein lysates were extracted from HCoEpiC cells or mouse colon tissue using pyrolysis buffer (Beyotime) and quantified and isolated on 10% SDS-PAGE gels (Invitrogen). Rabbit anti-human or mouse TLR2 (1:1000; Abcam), p65 (1:1000, Abcam, Cambridge, UK), P-p65 (1:1000, Abcam, Cambridge, UK) and GAPDH (1:2000, Abcam, Cambridge, UK) were used to imprint the protein onto a PVDF membrane (Millipore). HRP goat anti-rat IgG (1:20,000, Boster Biotechnology, Pleasanton, CA, USA) was incubated for 45 min as the secondary antibody. Strip visualization was performed by an enhanced chemiluminescence (ECL) system.

### RT‒PCR

Quantitative reverse transcriptase-PCR (qRT‒PCR) was used to assess the quantitative expression of *TLR2* and *CXCL1*. The sequences of the primers are listed in Supplementary Table 2. According to the manufacturer's instructions, total RNA was extracted from the tissues using TRIzol Reagent (Invitrogen). The OD_260_/OD_280_ ratio of total RNA extracted from HCoEpiC cells or colon tissue samples ranged from 1.8 to 2.0. Reverse transcription was performed in a 10 µL reaction volume using M-MLV reverse transcriptase (Takara, Japan) with 1 µg of RNA. Quantitative real-time PCR was performed using an ABI 7900 Detection System with SYBR Premix Ex TaqTM (Takara, Japan). Amplification included an initial denaturation step for 30 s at 95°C, followed by 40 cycles of PCR at 95°C for 5 s and at 60°C for 31 s, and a dissociation stage for 15 s at 95°C, 60 s at 60°C, and 15 s at 95°C. After the reactions were complete, the cycle threshold (CT) data were determined using fixed threshold settings, and the mean CT was determined from triplicate PCRs. A comparative CT method was used to compare each condition to the control reactions. mRNA levels were normalized to U6. The relative amount of gene normalized to the control was calculated with the Equation 2-△△CT.

### Enzyme-linked immunosorbent assay (ELISA)

The mouse blood samples were centrifuged, and serum was collected and immediately cryopreserved in liquid nitrogen. According to the manufacturer's instructions, the quantification of serum or cell culture supernatant cytokines was carried out using the GROα/CXCL1 (Growth Regulated Oncogene Alpha) ELISA Kit (Elabscience, Wuhan, China).

### Cell transfection

*CXCL1* and *TLR2* in HCoEpiC cells were inhibited by small interfering RNA (siRNA). siRNA was synthesized by Generalbiol Co. (Anhui, China). The sequences of the siRNAs are listed in Supplementary Table 3. siRNAs were transfected at a final concentration of 50 nM using Lipofectamine 2000 Transfection Reagent (GIBCO, 11668019) and Opti-MEM (Gibco, 31985070) reverse transfection protocols according to the manufacturer's instructions. RNA was recovered after 48 hours, and protein was recovered 72 hours after siRNA transfection.

### Immunofluorescence

Mouse liver tissues or neutrophils were fixed with 4% paraformaldehyde, stabilized in 0.2% Triton X-100 for 10 min until cell membrane rupture, washed with PBS 3 times, and immersed in 2% BSA for 30 min to inhibit nonspecific antigen binding sites. The tissues were then incubated with anti-MPO (1:300, Immunoway, Jiangsu, China, YM 6663) antibodies and anti-citH3 (1:500, Abcam, Cambridge, UK, ab5103) overnight at 4°C. The cells were then incubated with anti-MPO (1:300, Immunoway, Jiangsu, China, YM6663) antibodies or anti-citH3 (1:500, Abcam, Cambridge, UK, ab5103) overnight at 4°C. After washing, the tissues or cells were incubated with secondary antibody (Invitrogen) for 60 min, and the nuclei were stained with DAPI (Invitrogen) for 2 min. Then, the cells were washed with PBS and shielded from light before observation with a fluorescence microscope.

### Statistical Analysis

Statistics are presented as the mean ± standard deviation (SD). If the data were normally distributed, the t test was used for comparisons between groups. If the data were not normally distributed, differences were compared using the Mann‒Whitney U test. P < 0.05 was considered statistically significant.

### Availability of data and materials

The datasets used and/or analyzed during the current study are available from the corresponding author on reasonable request.

### Ethics approval and consent to participate

This study was approved by Ethical Committee of First Affiliated Hospital of Harbin Medical University. All animal experiments were approved by the Committee on the Ethics of Animal Experiments of First Affiliated Hospital of Harbin Medical University.

## AUTHOR CONTRIBUTION

WC Yao and YH He designed the study, analyzed the data and wrote the draft of the manuscript. ZH Xie, Q Wang and Y Chen prepared the figures and tables. JJ Yu and XX Liu assisted in the experiment. DB Xue, LY Wang and CJ Hao initiated the study, re-viewed and revised the manuscript. All authors read and approved the final manuscript.

## CONFLICT OF INTEREST

The authors have no relevant financial or non-financial interests to disclose.

## SUPPLEMENTAL MATERIAL

Click here for supplemental data file.

Click here for supplemental data file.

All supplemental data for this article are available online at www.microbialcell.com/researcharticles/2025a-yao-microbial-cell/.

## References

[1] Baiu I, Hawn MT (2018). Gallstones and Biliary Colic.. Jama.

[2] Lammert F, Gurusamy K, Ko CW, Miquel JF, Méndez-Sánchez N, Portincasa P, van Erpecum KJ, van Laarhoven CJ, Wang DQ (2016). Gallstones.. Nat Rev Dis Primers.

[3] Roa JC, García P, Kapoor VK, Maithel SK, Javle M, Koshiol J (2022). Gallbladder cancer.. Nat Rev Dis Primers.

[4] Hu H, Shao W, Liu Q, Liu N, Wang Q, Xu J, Zhang X, Weng Z, Lu Q, Jiao L, Chen C, Sun H, Jiang Z, Zhang X, Gu A (2022). Gut microbiota promotes cholesterol gallstone formation by modulating bile acid composition and biliary cholesterol secretion.. Nat Commun.

[5] Guan H, Zhang X, Kuang M, Yu J (2022). The gut-liver axis in immune remodeling of hepatic cirrhosis.. Front Immunol.

[6] Maurer KJ, Carey MC, Fox JG (2009). Roles of infection, inflammation, and the immune system in cholesterol gallstone formation.. Gastroenterology.

[7] Trivedi PJ, Adams DH (2016). Gut-liver immunity.. J Hepatol.

[8] Wiest R, Albillos A, Trauner M, Bajaj JS, Jalan R (2017). Targeting the gut-liver axis in liver disease.. J Hepatol.

[9] Grigor'eva IN, Romanova TI (2020). Gallstone Disease and Microbiome.. Microorganisms.

[10] Li G, Liu L, Lu T, Sui Y, Zhang C, Wang Y, Zhang T, Xie Y, Xiao P, Zhao Z, Cheng C, Hu J, Chen H, Xue D, Chen H, Wang G, Kong R, Tan H, Bai X, Li Z, McAllister F, Li L, Sun B (2023). Gut microbiota aggravates neutrophil extracellular traps-induced pancreatic injury in hypertriglyceridemic pancreatitis.. Nat Commun.

[11] Papayannopoulos V (2018). Neutrophil extracellular traps in immunity and disease.. Nat Rev Immunol.

[12] Muñoz LE, Boeltz S, Bilyy R, Schauer C, Mahajan A, Widulin N, Grüneboom A, Herrmann I, Boada E, Rauh M, Krenn V, Biermann MHC, Podolska MJ, Hahn J, Knopf J, Maueröder C, Paryzhak S, Dumych T, Zhao Y, Neurath MF, Hoffmann MH, Fuchs TA, Leppkes M, Schett G, Herrmann M (2019). Neutrophil Extracellular Traps Initiate Gallstone Formation.. Immunity.

[13] Chen H, Wang J, Ji Q, Jiang Z (2024). Sodium butyrate restricts neutrophils migration and NETs formation through reducing macrophage-derived CXCL16 in calculous cholecystitis.. Heliyon.

[14] Yoo W, Zieba JK, Foegeding NJ, Torres TP, Shelton CD, Shealy NG, Byndloss AJ, Cevallos SA, Gertz E, Tiffany CR, Thomas JD, Litvak Y, Nguyen H, Olsan EE, Bennett BJ, Rathmell JC, Major AS, Bäumler AJ, Byndloss MX (2021). High-fat diet-induced colonocyte dysfunction escalates microbiota-derived trimethylamine N-oxide.. Science.

[15] Zou J, Chassaing B, Singh V, Pellizzon M, Ricci M, Fythe MD, Kumar MV, Gewirtz AT (2018). Fiber-Mediated Nourishment of Gut Microbiota Protects against Diet-Induced Obesity by Restoring IL-22-Mediated Colonic Health.. Cell Host Microbe.

[16] Li Q, Hu W, Liu WX, Zhao LY, Huang D, Liu XD, Chan H, Zhang Y, Zeng JD, Coker OO, Kang W, Ng SSM, Zhang L, Wong SH, Gin T, Chan MTV, Wu JL, Yu J, Wu WKK (2021). Streptococcus thermophilus Inhibits Colorectal Tumorigenesis Through Secreting β-Galactosidase.. Gastroenterology.

[17] Teijeira Á, Garasa S, Gato M, Alfaro C, Migueliz I, Cirella A, de Andrea C, Ochoa MC, Otano I, Etxeberria I, Andueza MP, Nieto CP, Resano L, Azpilikueta A, Allegretti M, de Pizzol M, Ponz-Sarvisé M, Rouzaut A, Sanmamed MF, Schalper K, Carleton M, Mellado M, Rodriguez-Ruiz ME, Berraondo P, Perez-Gracia JL, Melero I (2020). CXCR1 and CXCR2 Chemokine Receptor Agonists Produced by Tumors Induce Neutrophil Extracellular Traps that Interfere with Immune Cytotoxicity.. Immunity.

[18] Chen Y, Wang Q, Gao W, Ma B, Xue D, Hao C (2021). Changes and Correlations of the Intestinal Flora and Liver Metabolite Profiles in Mice With Gallstones.. Front Physiol.

[19] Marion S, Studer N, Desharnais L, Menin L, Escrig S, Meibom A, Hapfelmeier S, Bernier-Latmani R (2019). In vitro and in vivo characterization of Clostridium scindens bile acid transformations.. Gut Microbes.

[20] Calton CM, Carothers K, Ramamurthy S, Jagadish N, Phanindra B, Garcia A, Viswanathan VK, Halpern MD (2024). Clostridium scindens exacerbates experimental necrotizing enterocolitis via upregulation of the apical sodium-dependent bile acid transporter.. Am J Physiol Gastrointest Liver Physiol.

[21] Leibovitzh H, Nayeri S, Borowski K, Hernandez-Rocha C, Lee SH, Turpin W, Stempak JM, Sandhu I, Milgrom R, Smith MI, Croitoru K, Hirschfield GM, Gulamhusein A, Silverberg MS (2024). Inflammatory bowel disease associated with primary sclerosing cholangitis is associated with an altered gut microbiome and bile acid profile.. J Crohns Colitis.

[22] Winkler ES, Shrihari S, Hykes Jr BL, Handley SA, Andhey PS, Huang YS, Swain A, Droit L, Chebrolu KK, Mack M, Vanlandingham DL, Thackray LB, Cella M, Colonna M, Artyomov MN, Stappenbeck TS, Diamond MS (2020). The Intestinal Microbiome Restricts Alphavirus Infection and Dissemination through a Bile Acid-Type I IFN Signaling Axis.. Cell.

[23] Burgess SL, Leslie JL, Uddin J, Oakland DN, Gilchrist C, Moreau GB, Watanabe K, Saleh M, Simpson M, Thompson BA, Auble DT, Turner SD, Giallourou N, Swann J, Pu Z, Ma JZ, Haque R, Petri Jr WA (2020). Gut microbiome communication with bone marrow regulates susceptibility to amebiasis.. J Clin Invest.

[24] Luissint AC, Fan S, Nishio H, Lerario AM, Miranda J, Hilgarth RS, Cook J, Nusrat A, Parkos CA (2024). CXADR-Like Membrane Protein Regulates Colonic Epithelial Cell Proliferation and Prevents Tumor Growth.. Gastroenterology.

[25] Chung L, Orberg ET, Geis AL, Chan JL, Fu K, DeStefano Shields CE, Dejea CM, Fathi P, Chen J, Finard BB, Tam AJ, McAllister F, Fan H, Wu X, Ganguly S, Lebid A, Metz P, Van Meerbeke SW, Huso DL, Wick EC, Pardoll DM, Wan F, Wu S, Sears CL, Housseau F (2018). Bacteroides fragilis Toxin Coordinates a Pro-carcinogenic Inflammatory Cascade via Targeting of Colonic Epithelial Cells.. Cell Host Microbe.

[26] Tsoi H, Chu ESH, Zhang X, Sheng J, Nakatsu G, Ng SC, Chan AWH, Chan FKL, Sung JJY, Yu J (2017). Peptostreptococcus anaerobius Induces Intracellular Cholesterol Biosynthesis in Colon Cells to Induce Proliferation and Causes Dysplasia in Mice.. Gastroenterology.

[27] Xie D, Han C, Chen C, Liao Z, Campos de Souza S, Niu Y, Mano JF, Dong L, Wang C (2024). A scaffold vaccine to promote tumor antigen cross-presentation via sustained toll-like receptor-2 (TLR2) activation.. Bioact Mater.

[28] Haque M, Kaminsky L, Abdulqadir R, Engers J, Kovtunov E, Rawat M, Al-Sadi R, Ma TY (2024). Lactobacillus acidophilus inhibits the TNF-α-induced increase in intestinal epithelial tight junction permeability via a TLR-2 and PI3K-dependent inhibition of NF-κB activation.. Front Immunol.

[29] Janeesh PA, Sasikala V, Dhanya CR, Abraham A (2014). Robinin modulates TLR/NF-κB signaling pathway in oxidized LDL induced human peripheral blood mononuclear cells.. Int Immunopharmacol.

[30] Kawai T, Akira S (2007). Signaling to NF-kappaB by Toll-like receptors.. Trends Mol Med.

[31] Liu B, Fan L, Wang Y, Wang H, Yan Y, Chen S, Hung I, Liu C, Wei H, Ge L, Ren W (2024). Gut microbiota regulates host melatonin production through epithelial cell MyD88.. Gut Microbes.

[32] Liu X, Tang R, Xu J, Tan Z, Liang C, Meng Q, Lei Y, Hua J, Zhang Y, Liu J, Zhang B, Wang W, Yu X, Shi S (2023). CRIP1 fosters MDSC trafficking and resets tumour microenvironment via facilitating NF-κB/p65 nuclear translocation in pancreatic ductal adenocarcinoma.. Gut.

[33] Kemp SB, Carpenter ES, Steele NG, Donahue KL, Nwosu ZC, Pacheco A, Velez-Delgado A, Menjivar RE, Lima F, The S, Espinoza CE, Brown K, Long D, Lyssiotis CA, Rao A, Zhang Y, Pasca di Magliano M, Crawford HC (2021). Apolipoprotein E Promotes Immune Suppression in Pancreatic Cancer through NF-κB-Mediated Production of CXCL1.. Cancer Res.

[34] Li Y, Chen L, Sottas C, Raul MC, Patel ND, Bijja JR, Ahmed SK, Kapelanski-Lamoureux A, Lazaris A, Metrakos P, Zambidis A, Chopra S, Li M, Sugahara G, Saito T, Papadopoulos V (2024). The mitochondrial TSPO ligand Atriol mitigates metabolic-associated steatohepatitis by downregulating CXCL1.. Metabolism.

[35] Crespo J, Wu K, Li W, Kryczek I, Maj T, Vatan L, Wei S, Opipari AW, Zou W (2018). Human Naive T Cells Express Functional CXCL8 and Promote Tumorigenesis.. J Immunol.

[36] Krishnamurthy A, Joshua V, Haj Hensvold A, Jin T, Sun M, Vivar N, Ytterberg AJ, Engström M, Fernandes-Cerqueira C, Amara K, Magnusson M, Wigerblad G, Kato J, Jiménez-Andrade JM, Tyson K, Rapecki S, Lundberg K, Catrina SB, Jakobsson PJ, Svensson C, Malmström V, Klareskog L, Wähämaa H, Catrina AI (2016). Identification of a novel chemokine-dependent molecular mechanism underlying rheumatoid arthritis-associated autoantibody-mediated bone loss.. Ann Rheum Dis.

[37] Xiong G, Chen Z, Liu Q, Peng F, Zhang C, Cheng M, Ling R, Chen S, Liang Y, Chen D, Zhou Q (2024). CD276 regulates the immune escape of esophageal squamous cell carcinoma through CXCL1-CXCR2 induced NETs.. J Immunother Cancer.

[38] Hilscher MB, Sehrawat T, Arab JP, Zeng Z, Gao J, Liu M, Kostallari E, Gao Y, Simonetto DA, Yaqoob U, Cao S, Revzin A, Beyder A, Wang RA, Kamath PS, Kubes P, Shah VH (2019). Mechanical Stretch Increases Expression of CXCL1 in Liver Sinusoidal Endothelial Cells to Recruit Neutrophils, Generate Sinusoidal Microthombi, and Promote Portal Hypertension.. Gastroenterology.

[39] Antipenko S, Mayfield N, Jinno M, Gunzer M, Ismahil MA, Hamid T, Prabhu SD, Rokosh G (2024). Neutrophils are indispensable for adverse cardiac remodeling in heart failure.. J Mol Cell Cardiol.

[40] Pan JJ, Xie SZ, Zheng X, Xu JF, Xu H, Yin RQ, Luo YL, Shen L, Chen ZR, Chen YR, Yu SZ, Lu L, Zhu WW, Lu M, Qin LX (2024). Acetyl-CoA metabolic accumulation promotes hepatocellular carcinoma metastasis via enhancing CXCL1-dependent infiltration of tumor-associated neutrophils.. Cancer Lett.

[41] Chen KW, Monteleone M, Boucher D, Sollberger G, Ramnath D, Condon ND, von Pein JB, Broz P, Sweet MJ, Schroder K (2018). Noncanonical inflammasome signaling elicits gasdermin D-dependent neutrophil extracellular traps.. Sci Immunol.

[42] Hisada Y, Grover SP, Maqsood A, Houston R, Ay C, Noubouossie DF, Cooley BC, Wallén H, Key NS, Thålin C, Farkas Á Z, Farkas VJ, Tenekedjiev K, Kolev K, Mackman N (2020). Neutrophils and neutrophil extracellular traps enhance venous thrombosis in mice bearing human pancreatic tumors.. Haematologica.

[43] Raup-Konsavage WM, Wang Y, Wang WW, Feliers D, Ruan H, Reeves WB (2018). Neutrophil peptidyl arginine deiminase-4 has a pivotal role in ischemia/reperfusion-induced acute kidney injury.. Kidney Int.

[44] Huang H, Tohme S, Al-Khafaji AB, Tai S, Loughran P, Chen L, Wang S, Kim J, Billiar T, Wang Y, Tsung A (2015). Damage-associated molecular pattern-activated neutrophil extracellular trap exacerbates sterile inflammatory liver injury.. Hepatology.

[45] Quast C, Pruesse E, Yilmaz P, Gerken J, Schweer T, Yarza P, Peplies J, Glöckner FO (2013). The SILVA ribosomal RNA gene database project: improved data processing and web-based tools.. Nucleic Acids Res.

[46] Kõljalg U, Nilsson RH, Abarenkov K, Tedersoo L, Taylor AF, Bahram M, Bates ST, Bruns TD, Bengtsson-Palme J, Callaghan TM, Douglas B, Drenkhan T, Eberhardt U, Dueñas M, Grebenc T, Griffith GW, Hartmann M, Kirk PM, Kohout P, Larsson E, Lindahl BD, Lücking R, Martín MP, Matheny PB, Nguyen NH, Niskanen T, Oja J, Peay KG, Peintner U, Peterson M (2013). Towards a unified paradigm for sequence-based identification of fungi.. Mol Ecol.

[47] Parks DH, Tyson GW, Hugenholtz P, Beiko RG (2014). STAMP: statistical analysis of taxonomic and functional profiles.. Bioinformatics.

[48] Langille MG, Zaneveld J, Caporaso JG, McDonald D, Knights D, Reyes JA, Clemente JC, Burkepile DE, Vega Thurber RL, Knight R, Beiko RG, Huttenhower C (2013). Predictive functional profiling of microbial communities using 16S rRNA marker gene sequences.. Nat Biotechnol.

[49] Ren Z, Li A, Jiang J, Zhou L, Yu Z, Lu H, Xie H, Chen X, Shao L, Zhang R, Xu S, Zhang H, Cui G, Chen X, Sun R, Wen H, Lerut JP, Kan Q, Li L, Zheng S (2019). Gut microbiome analysis as a tool towards targeted non-invasive biomarkers for early hepatocellular carcinoma.. Gut.

[50] Li L, Jing Q, Yan S, Liu X, Sun Y, Zhu D, Wang D, Hao C, Xue D (2021). Amadis: A Comprehensive Database for Association Between Microbiota and Disease.. Front Physiol.

