# Supplementary material for: Clostridium scindens promotes gallstone formation by inducing intrahepatic neutrophil extracellular traps through CXCL1 produced by colonic epithelial cells
Source: Microb Cell. 2025 Mar 19;12:37–52. doi: 10.15698/mic2025.03.844 (PMC12041793; doi:10.15698/mic2025.03.844)
Supplement: Supplementary file 2 [file mic-12-037-s02.pdf]

Table 1. Sequence of Primer designed by Generalbiol Co.

PIN	Primer Name	Sequence (5′ to 3′)	Length	Tm℃	GC %	Purification
D568106	(Mus)CXCL1-F	ACTGCACCCAAACCGA AGTC	20	54.4	55	RPC
D568107	(Mus)CXCL1-R	TGGGGACACCTTTTAG CATCTT	22	53.6	45.5	RPC
D568108	(Mus)TLR2-F	CTCTTCAGCAAACGCT GTTCT	21	52.4	47.6	RPC
D568109	(Mus)TLR2-R	GGCGTCTCCCTCTATTG TATTG	22	55.4	50	RPC
D096310	(Homo)TLR2-F	ACATTAGCAACAGTGA CCTACAGA	24	54.0	41.7	RPC
D096311	(Homo)TLR2-R	GCTTGAACCAGGAAGA CGATA	21	52.4	47.6	RPC
D096312	(Homo)CXCL1-F	ACCCCAAGAACATCCA AAGT	20	50.4	45	RPC
D096313	(Homo)CXCL1-R	GATGCAGGATTGAGGC AAG	19	51.5	52.6	RPC

Table 2. Sequence of siRNA-TLR2 and CXCL1 designed by Generalbiol Co.

Primer Name	Sequence (5′ to 3′)	Length
TLR2 (human) siRNA-1	AGUUAUAGAUCCAGGUAAATT	21
	UUUACCUGGAUCUAUAACUTT	21
TLR2 (human) siRNA-2	UCUUAAACUUACUGGGAAATT	21
	UUUCCCAGUAAGUUUAAGATT	21
**TLR2 (human) siRNA-3**	CGGGAAGGAUUUUGGGUAATT	21
	UUACCCAAAAUCCUUCCCGTT	21
CXCL1 (human) siRNA-239	CCAAGAACAUCCAAAGUGUTT	21
	ACACUUUGGAUGUUCUUGGTT	21
CXCL1 (human) siRNA-293	CCGAAGUCAUAGCCACACUTT	21
	AGUGUGGCUAUGACUUCGGTT	21
**CXCL1 (human) siRNA-319**	GGGCGGAAAGCUUGCCUCATT	21
	UGAGGCAAGCUUUCCGCCCTT	21
